# Pharyngeal motility in patients submitted to type I thyroplasty

**DOI:** 10.1016/j.bjorl.2019.11.007

**Published:** 2019-12-18

**Authors:** Bruno Rezende Pinna, Fernando A.M. Herbella, Noemi de Biase

**Affiliations:** a Universidade Federal de São Paulo, Escola Paulista de Medicina, Departamento de Otorrinolaringologia, São Paulo, SP, Brazil; b Universidade Federal de São Paulo, Escola Paulista de Medicina, Departamento de Cirurgia, São Paulo, SP, Brazil; c Pontifícia Universidade Católica de São Paulo, São Paulo, SP, Brazil

**Keywords:** Upper esophageal sphincter, Deglutition disorders, High resolutionmanometry, Vocal fold immobility

## Abstract

**Introduction:**

Type 1 thyroplasty is performed to improve glottis closure as well as dysphagic symptoms in patients with unilateral vocal fold immobility.

**Objectives:**

This study aims to compare the motility of the pharynx and upper esophageal sphincter in patients with unilateral vocal fold immobility before and after thyroplasty Type I.

**Methods:**

We prospectively studied 15 patients with unilateral vocal fold immobility who underwent thyroplasty Type I. Subjects were divided according to the topography of vagal injury and presence of dysphagia. High resolution manometry was performed before and 30 days after surgery. Time and pressure manometric parameters at the topography of the velopharynx, epiglottis and upper esophageal sphincter were recorded.

**Results:**

Dysphagia was present in 67% of patients. 63% had lower vagal injuries. Manometric parameters did not change after thyroplasty for the whole population. The group of dysphagic patients, however, had an increase in residual pressure at the upper esophageal sphincter after thyroplasty (1.2 vs. 5.2 mmHg; *p* = 0.05). Patients with low vagal injury developed higher peak pressure (100 vs. 108.9 mmHg *p* ≤ 0.001), lower rise time (347 vs. 330 ms *p* = 0.04), and higher up stroke (260 vs. 266.2 mmHg/ms *p* = 0.04) at the topography of the velopharynx after thyroplasty.

**Conclusion:**

Pharyngeal motility is affected by thyroplasty Type I in patients with dysphagia and low vagal injury.

## Introduction

Swallowing and prevention of aspiration are complex acts that encompass over 30 muscles and 6 cranial nerves.^1^ Vagal integrity is essential to these acts since up to 40% of patients with unilateral vocal fold immobility (UVFI) have some degree of aspiration^2^, ^3^ and 6% of patients with dysphagia may have UVFI as the etiology.^4^

Dysphagia after vagal paralysis is multifactorial and may be linked to: (a) loss of sensation via the internal branch of the superior laryngeal nerve, (b) loss of the vagal contribution to the pharyngeal plexus and (c) vocal fold incompetence caused by injuries of the recurrent laryngeal nerve.^5^

High resolution manometry (HRM) was recently added to the armamentarium for the study of the pharynx and proximal esophagus due to technological advances in comparison to conventional manometry, such as the rapid response of solid state sensors, circumferential disposition of sensors and compensation for motion artifacts.^6^

Previous studies have shown that pharyngeal motility is affected in patients with UVFI.^7^, ^8^ Vocal fold medialization (VFM) seems to improve dysphagia symptoms in patients with UVFI.^5^ Both injection medialization and Type I thyroplasty have been found to improve rates of penetration and aspiration on fluoroscopy^8^ and bring better quality of life to dysphagic patients.^9^ However, whether VFM has any kind of influence on pharyngeal motility and if the improvement in dysphagia symptoms after VFM is related to changes in pharyngeal motility is still elusive.

This study aims to compare the motility of the pharynx in patients with UVFI before and after Type I thyroplasty.

## Methods

### Population

We prospectively studied 15 patients, median age 50 (32–59) years, 10 (75%) females, with peripheral vagal injury, submitted to Type I thyroplasty. Subjects were divided according to the topography of vagal injury and the presence of dysphagia. We considered High Vagal Injuries (HVI) those patients with lesion of pharyngeal branch and Low Vagal Injuries (LVI) patients with lesions of the recurrent or superior laryngeal nerves. Topography determination was made by clinical criteria considering the etiology of UVFI and by nasofibrolaryngoscopy where it was possible to see pharynx movement. No EMG was performed. HVI (pharyngeal injury) occurred in 4 patients (26.7%) due to glomus tumor resection. According to ENT exams, IX cranial nerve injury was present in 4 patients and XI cranial nerve injury was present in one patient. LVI was present in 11 (7 post-total thyroidectomy, 2 after spine surgery, 1 due to aortic arch aneurysm and 1 after thoracic gunshot.

Patients were excluded from the study in the following conditions: inability to undergo a HRM, inability to understand or comply with the protocol or those who refused to sign the informed consent.

### Otolaryngologic workup

All patients were originally evaluated in the Ear, Nose and Throat clinic due to suspected vagal injury based on complaints suggesting vocal impairment, and/or dysphagia.

All patients underwent a complete nasofibro- laryngoscopy and flexible endoscopic evaluation of swallowing (FEES).^10^ Initially patients were observed with flexible endoscopy during resting breathing. Swallow function was examined while patients swallowed two trials each of honey thick liquid (5 and 10 mL), nectar thick liquid (5 and 10 mL), thin liquid (5, 10 and 90 mL), puree (5 mL) and solid food (1/2 saltine cracker). If gross aspiration was seen with any viscosity of liquid, the clinician did not continue to other consistencies and moved to puree; if aspiration persisted, a clinical decision was made to stop the exam. As all patients with HVL had aspiration with liquid and puree, FEES parameters used to compare HVL and LVL patients were only puree and liquid. Vagal injury was confirmed by unilateral vocal cord paralysis.

Dysphagia was evaluated based on FEES and self-reported swallowing disability. This was assessed with the SWAL QOL^11^ (Swallowing Questionnaire of Life) questionnaire before and after surgery.

### High resolution manometry

HRM was performed as previously described.^6^ In summary, a 36 sensors 1 cm spaced system (Medtronic, Minneapolis) was used. After a 20 second period to assess Upper Esophageal Sphincter (UES) basal pressure, patients were given ten swallows of 5 mL of water. Sterile water was presented via syringe, and subjects held the bolus in the oral cavity until cued to swallow. Small bolus size of 2 mL was used in subjects known to be aspirators. The patient was kept in upright position. Dedicated software (ManoScan and Manoview 3.0, Medtronic, Minneapolis) was used for manometric data acquisition and analysis. Non-conventional measurements were performed with manual and individual adjustments of the software tools. The same experienced investigator did all the tests and analysis.

Time and pressure manometric variables at the topography of the velopharynx (VP), epiglottis and UES were recorded. The following manometric parameters were studied:

At the topography of the velopharynx and epiglottis:

Peak pressure: measured at the point of highest pressure (mmHg);

Rise time: time interval from onset of pharyngeal contraction to peak pressure (ms);

Upstroke: peak pressure/rise time rate (mmHg/ms);

Recovery time: time interval from peak pressure to the end of the contraction (ms);

Contraction duration (ms).

At the topography of the UES:

Extension (cm);

Basal pressure: measured at the midpoint, during the 20 seconds period before the first swallow (mmHg);

Residual pressure: nadir (lowest) pressure at the midpoint (mmHg);

Relaxation duration: (ms);

Relaxation time to nadir: time between the beginning of the relaxation and the nadir pressure (ms);

Recovery time: time between nadir and the end of the relaxation (ms).

Normal values were based on previous studies in volunteers.^6^ HRM was performed before surgery and 30 days after the procedure.

### Type I thyroplasty

Laryngeal framework surgery was performed in all patients with sedation and local anesthesia. A window in the thyroid cartilage was created immediately to the level of the affected vocal fold. A silicone implant was hand carved with a scalpel and placed into the laryngoplasty window without the need for suture fixation. The point of maximal displacement and depth of medialization were achieved by the aid of vocal tasks. The median duration of UVFI from symptom onset to date of surgery was 13 months, with the shortest duration recorded as 8 months. All patients underwent speech therapy before surgery.

### Statistics

Data is shown as median (interquartile range). All data, except basal pressure, correspond to a mean of 10 swallows.

Shapiro–Wilk test was used to assess normal distribution of data. Non-parametric tests were selected and medians were compared by the Mann–Whitney test due to the small number of patients and non-normal distribution of most variables; *p* < 0.05 was considered significant.

### Ethics

The study protocol was approved by the local Ethics Committee (1.979.860) and written informed consent was obtained from each subject.

There is no conflict of interest. All authors contributed sufficiently to be named as authors and are responsible for the manuscript. No professional or ghost writer was hired.

## Results

### Otolaryngologic parameters

Vocal cord paralysis was more frequent on the left side (62% vs. 37%).

**Figure 1:**
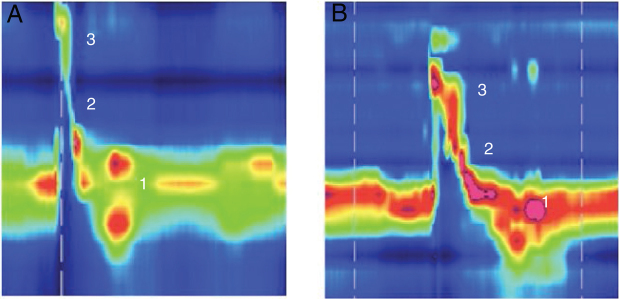
Example of high resolution manometry before and after Type I thyroplasty showing increase in peak pressure in the velopharynx area, in a patient with low vagal injury (vocal fold paralysis after thyroid surgery). (1) UES; (2) Epiglottis; (3) Velopharynx. A, Pre surgery; B, Pos surgery.

**Figure 2:**
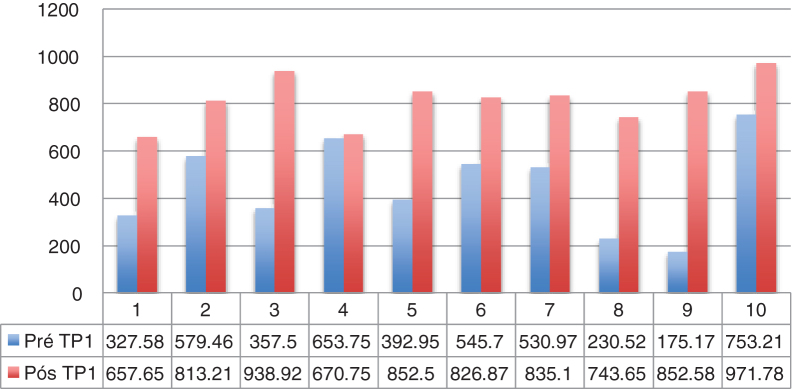
SWAL QOl score for each dysphagic patient (n = 10). HVL, Patients 1, 5, 7, 9; LVL, Patients 2, 3, 4, 6, 8, 10 (raised in reviewer comments).

Dysphagia was present in 10 (67%) patients (Table 1). All patients HVL lesions had dysphagia. Aspiration was present in 4 patients (27%) and only in patients with high vagal lesions. Among 11 patients with LVL, 6 (54.5%) had dysphagia. All dysphagic patients reported improvement after thyroplasty. There was an improvement in SWAL QOL global score for dysphagic population as a whole; Pre 431.97 vs. Pos 830.98 (*p* < 0.05) (Table 2). The SWAL QOl result for each dysphagic patient is represented in and Figure 1, Figure 2.

**Table 1:** FESS findings in dysphagic patients before Type I thyroplasty (n = 10).

	Bolus retention		Laryngeal penetration		Aspiration	
	(%)	(n)	(%)	(n)	(%)	(n)
Puree bolus consistency						
LVL (n = 6)	100	6	33	2	0	0
HVL (n = 4)	100	4	100	4	100	4
Liquid bolus consistency						
LVL (n = 6)	50	3	50	3	0	0
HVL (n = 4)	100	4	100	4	100	4

HVL, High vagal lesions (n = 4); LVL, Low vagal lesions (n = 6).

**Table 2:** SWAL QOL findings in the whole dysphagic population before and after Type I thyroplasty (n = 10).

	Pre surgery	After surgery	*p*
Swal Qol	431.97 (335‒571)	830 (761–852)	(0.0004)

### Manometric parameters

Manometric parameters did not change before and after Type I thyroplasty for the whole population (Table 3).

**Table 3:** Manometric findings before and after Type I thyroplasty in all patients (n = 15).

	**Velopharynx**			**Epiglotis**		
	Preoperative	Postoperative	*p*	Preoperative	Postoperative	*p*
Peak pressure (mmHg)	77 (26–102)	99 (42–127)	0.3	96 (43–115)	124 (53–145)	0.1
Rise time (ms)	345 (282–391)	354 (278–424)	1	301 (282–337)	284 (266–308)	0.2
Upstroke (mmHg/ms)	199 (112–292)	243 (78–296)	0.8	239 (11–339)	366 (154–500)	0.2
Recovery time (ms)	304 (202–463)	320 (240–489)	0.8	225 (182–292)	260 (225–338)	0.5
Contraction duration (ms)	678 (526–848)	695 (526–848)	0.5	526 (474–587)	556 (506–690)	1
	**Upper esophageal sphincter**					
Basal pressure (mmHg)	58.3 (29.1–85.9)	46.6 (31–79)	0.9			
Residual pressure (mmHg)	3.2 (0.1–3.2)	5.1 (3.8–7.45)	0.07			
Relaxation time to nadir (ms)	235 (188–297)	239 (180–261)	0.8			
Relaxation duration (ms)	660 (615–750)	674 (514–739)	0.5			
Recovery time (ms)	374 (581‒325)	434 (321–490)	0.7			
Extension (cm)	3 (3‒3)	3 (3‒3)	0.7			

The group of patients with dysphagia before operation had no manometric changes at the topography of pharynx after surgery. However an increase in residual pressure at the UES after thyroplasty was observed (1.7 vs. 5.1 mmHg; *p* = 0.02) (Table 4).

**Table 4:** Manometric findings before and after Type I thyroplasty in patients with preoperative dysphagia (n = 10).

	**Velopharynx**			**Epiglotis**		
	Preoperative	Postoperative	*p*	Preoperative	Postoperative	*p*
Peak pressure (mmHg)	56 (21–113)	96 (34–148)	0.4	57 (9–103)	76 (19–123)	0.4
Rise time (ms)	298 (260–329)	276 (257–330)	0.5	361 (344–441)	353 (270–426)	0.4
Upstroke (mmHg/ms)	143 (81–314)	278 (97–504)	0.3	167 (49–262)	142 (57–279)	0.7
Recovery time (ms)	269 (216–294)	272 (243–351)	0.4	340 (258–501)	373 (232–516)	0.5
Contraction duration (ms)	578 (512–587)	519 (519–744)	0.3	749 (609–875)	753 (530–882)	0.6
	**Upper esophageal sphincter**					
Basal pressure (mmHg)	46 (23–79)	46 (23–68)	0.8			
Residual pressure (mmHg)	1 (0–3)	5 (4–8)	0.02*			
Relaxation time to nadir (ms)	248 (207–331)	241 (208–264)	0.8			
Relaxation duration (ms)	676 (614–851)	641 (537–710)	0.6			
Recovery time (ms)	365 (315–484)	356 (319–456)	0.7			
Extension (cm)	3 (3‒3)	3 (3‒3)	1			

* Statiscally significant.

The comparison of manometric parameters according to the topography of vagal injury is depicted in Table 5, Table 6. Patients with LVI developed higher peak pressure (100 vs. 108.9 mmHg; *p* = 0.004); lower rise time (347 vs. 330 ms; *p* = 0.04); and higher up-stroke (260 vs. 266.2 mmHg/ms; *p* = 0.0007) at the topography of the velopharynx after thyroplasty (Table 5). Manometric parameters did not change after thyroplasty in patients with HVI (Table 6).

**Table 5:** Manometric findings before and after Type I thyroplasty in patients with low vagal injury (n = 11).

	**Epiglotis**			**Velopharynx**		
	Preoperative	Postoperative	*p*	Preoperative	Postoperative	*p*
Peak pressure (mmHg)	112 (99–117)	131 (125–153)	0.1	100 (77–112)	108 (85–128)	0.0004*
Rise time (ms)	305 (289–337)	284 (274–292)	0.09	347 (291–395)	330 (271–444)	0.004*
Upstroke (mmHg/ms)	316 (247–355)	388 (324–521)	0.1	260 (184–293)	266.2 (182–300)	0.0007*
Recovery time (ms)	213 (160–284)	258 (234–277)	0.3	316 (235–581)	296 (256–407)	0.5
Contraction duration (ms)	515 (466–582)	550 (519–650)	0.3	725 (556–880)	657 (564–782)	0.7
	**Upper esophageal sphincter**					
Basal pressure (mmHg)	64 (39–89)	69 (29–90)	0.8			
Residual pressure (mmHg)	3 (1–6)	5 (3–6)	0.6			
Relaxation time to nadir (ms)	247 (182–302)	234 (179–264)	0.6			
Relaxation duration (ms)	669 (638–726)	688 (525–752)	0.8			
Recovery time (ms)	392 (285–555)	460 (346–512)	0.7			
Extension (cm)	3 (3‒3)	3 (3‒3)	1			

* Statiscally significant.

**Table 6:** Manometric findings before and after Type I thyroplasty in patients with high vagal injury (n = 4).

	**Velopharynx**			**Epiglotis**		
	Preoperative	Postoperative	*p*	Preoperative	Postoperative	*p*
Peak pressure (mmHg)	6 (5–9)	12 (8–64)	0.3	15 (11–21)	18 (9–47)	0.8
Rise time (ms)	312 (281–350)	325 (291–354)	0.7	268 (239–305)	259 (246–283)	1
Upstroke (mmHg/ms)	28 (12–120)	26.(15–226)	0.6	69 (54–81)	63 (40–191)	0.8
Recovery time (ms)	212 (176–270)	369 (190–605)	0.4	285 (258–359)	220 (115–397)	0.8
Contraction duration (ms)	524 (458–621)	694 (481–959)	0.7	548 (491–664)	503 (345–721)	0.8
	**Upper esophageal sphincter**					
Basal pressure (mmHg)	25 (13–42)	46 (39–51)	0.09			
Residual pressure (mmHg)	−1 (−3–1)	6. (0–11)	0.3			
Relaxation time to nadir (ms)	233 (183–281)	220 (173–283)	0.8			
Relaxation duration (ms)	583 (536–698)	551 (441–630)	0.6			
Recovery time (ms)	364 (352–431)	331 (268–346)	0.1			
Extension (cm)	3 (3–3)	3 (3–3)	1			

## Discussion

Previous reports with video fluoroscopic swallowing studies (VFSS) showed pharyngeal motility impairment in patients with UVFI. Bhattacharyya et al.^8^ detected pharyngeal phase abnormalities such as delayed initiation of swallowing, reduced laryngeal elevation, and reduced upper ssophageal Sphincter (UES) opening in these patients. Domer et al.^12^ demonstrated that individuals with either idiopathic or iatrogenic UVFI had prolonged total pharyngeal transit time and elevated pharyngeal constriction rates, suggesting delayed bolus transit and pharyngeal weakness.

HRM replaced conventional manometry for the study of esophageal motility due to a more intuitive interpretation of data, improved comfort to the patients and the possibility of more accurate measurements and new metrics.^13^ At the level of the pharynx, HRM contribution is still incipient. The first study of the pharynx in swallowing disorders, not only normal physiology in volunteers, is a case report published in 2010.^14^ Currently, sophisticated and complex time and pressure parameters were determined at the pharynx at light of HRM.^6^ Most of them are yet to be validated for use in clinical practice.

There are few studies regarding vocal fold immobility and manometry. Wilson et al.^15^ studied 27 patients and 25 healthy volunteers as controls with solid-state conventional manometry. Interestingly, the authors found different patterns of motility according to the anatomical vagal injury, with UES compromised in central lesions and pharynx in peripheral lesions. We have also previously observed that pharyngeal motility is significantly impaired in UVFI patients. One-third showed a hypotonic sphincter; vellum pressure was hypotonic in half of patients. The subgroup of dysphagic patients had lower residual UES pressure and lower epiglottis peak pressure as compared to non-dysphagic patients.^7^

In the current study, pharyngo-upper esophageal motility was unaltered by the surgical procedure, when considering the whole population. Pharyngeal motility is affected by thyroplasty Type I only in patients with dysphagia and low vagal injury.

This study evaluated the self-reported symptoms of dysphagia. The SWAL QOl does not have a threshold score and a normative data. The scores were used in order to state the improvement in swallowing disorders in the dysphagic population after Type I thyroplasty. Previous studies have already shown the benefits of vocal fold medialization in this this population.^5^, ^9^ Despite the fact that all patients reported improvement in clinical symptoms, and SWAL QOL score increased in the population as a whole, no correlation was made between the questionnaire and HRM parameters. Based on our findings we can not affirm that dysphagia improvement is related to any change in pharyngeal motility.

### Upper esophageal sphincter and thyroplasty

UES residual pressure had an increase after thyroplasty in the subanalysis of the individuals with dysphagia before the operation. Other UES parameters were unaltered.

Previous studies have shown that UES is kept shut by the tonic contraction of the cricopharyngeus muscle^16^ and relaxation occurs by the action of 3 major combined factors : relaxation of the cricopharyngeus muscle, contractions of the suprahyoid and thyrohyoid muscles to move the larynx to the front, and the hydrostatic pressure by the bolus.^17^ Type I thyroplasty, itself, does not directly affect any of these three major mechanisms to explain the reason for the increase in residual pressure, and specifically why it occurred only in dysphagic patients. Although UES residual pressure changed after the operation it remained within normal values range. The average value was close to the inferior range of normality before the operation and raised to values close to the superior threshold. Despite the fact that all patients with dysphagia before the operation reported improvement in clinical symptoms, as stated by the SWAL QOL, we cannot affirm if the increase in residual pressure is the reason for to the improvement in swallowing functions and we cannot state a relation between clinical status and HRM findings. We believe that this clinical improvement in dysphagia is more related to a better glottal efficiency achieved by VFM rather than any changes occurred in the UES, as Type I thyroplasty does not cause any kind of anatomic or structural changes in the UES area. We should emphasize that studies regarding pharynx procedures HRM and UES are still very incipient. Further studies in this area will surely produce more elucidative conclusions about the topic.

### Velopharynx and thyroplasty

In the low vagal lesion group, we found an increase in peak pressure and upstroke and a decrease in rise time in the velopharynx area.

Low subglottic air pressure can worsen the swallowing-breathing interaction in UVFI patients and increase swallowing disorders because of a damaged airway protection mechanism.^18^ VFM can lead to an increase in the subglottic pressure. We hypothesize that this change in subglottic pressure may be the reason for the increase in peak pressure at the velopharynx after Type I thyroplasty. This phenomenon occurred only in patients with low vagal injury. Velopharynx pressurization is accomplished through the action of different muscles, including the palatopharyngeus, palatoglossus, and superior pharyngeal constrictors. Its innervation is mostly achieved by the pharyngeal branch of the vagus nerve^19^ that is preserved in those patients with low vagal injuries. This increase was not observed in patients with high vagal injuries, as the pharyngeal branch was damaged in this situation (Fig. 1).

The decrease in rise time has probably the same explanation as to why peak pressure is increased. The activation of pharyngeal muscles results in a decreased time to achieve a higher peak pressure. The increase in upstroke is a consequence of the increase in peak pressure and the decrease in rise time as upstroke is the relation between the two variables.

### Epiglottis and thyroplasty

We did not find changes in epiglottis area in any of the subgroups studied. Measuring velopharynx pressure is more sensitive than measuring epiglottis pressure for demonstrating pharyngeal pressure because of the anatomically narrow cone shape of velopharynx structure compared to the wide and irregularly cylindrical shape of epiglottis.^17^ Furthermore the epiglottis area also reflects tongue base pressures,^6^ an area that has little innervation by the vagus nerve.

Our study has some limitations. This study comprises a very small number of patients and all patients volunteered for the study since HRM is not yet part of the clinical care of these individuals. This small number of patients also precluded a subanalysis of different dysphagia degrees. The 30 days between tests was chosen in order to prevent any influence from speech therapy among preoperative dysphagic patients in HRM parameters.

## Conclusions

In conclusion, dysphagia improvement in patients submitted to Type I thyroplasty is probably not related to changes in pharyngeal motility. However, pharyngeal motility is affected by Type I thyroplasty in a subset of patients. These changes were more pronounced in dysphagic patients and patients with low vagal injuries.

## Conflicts of interest

The authors declare no conflicts of interest.
